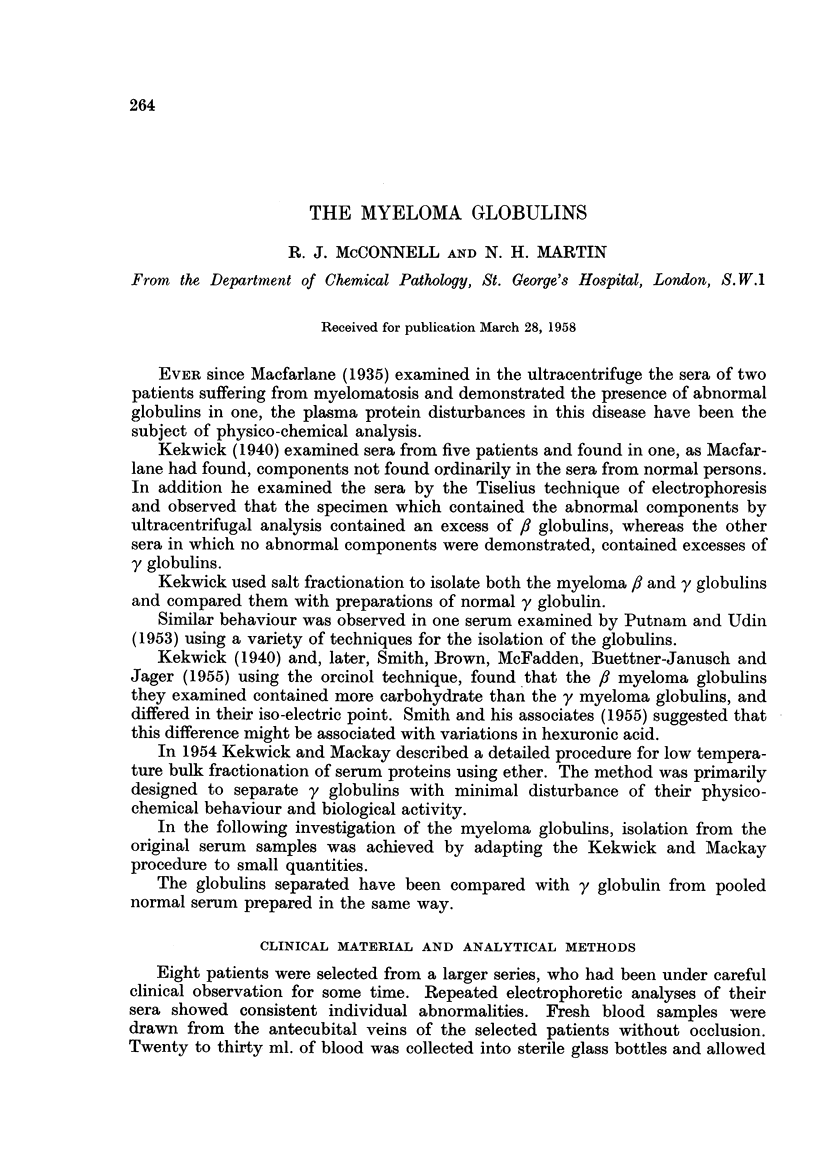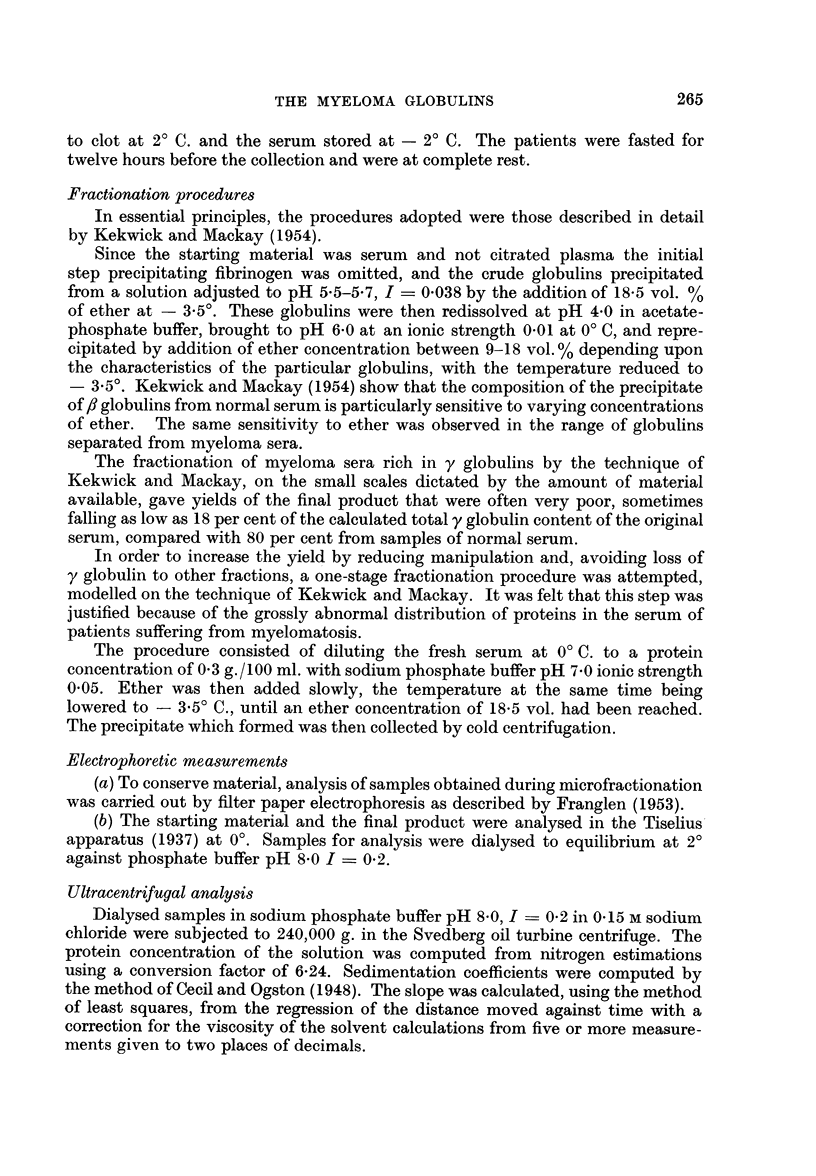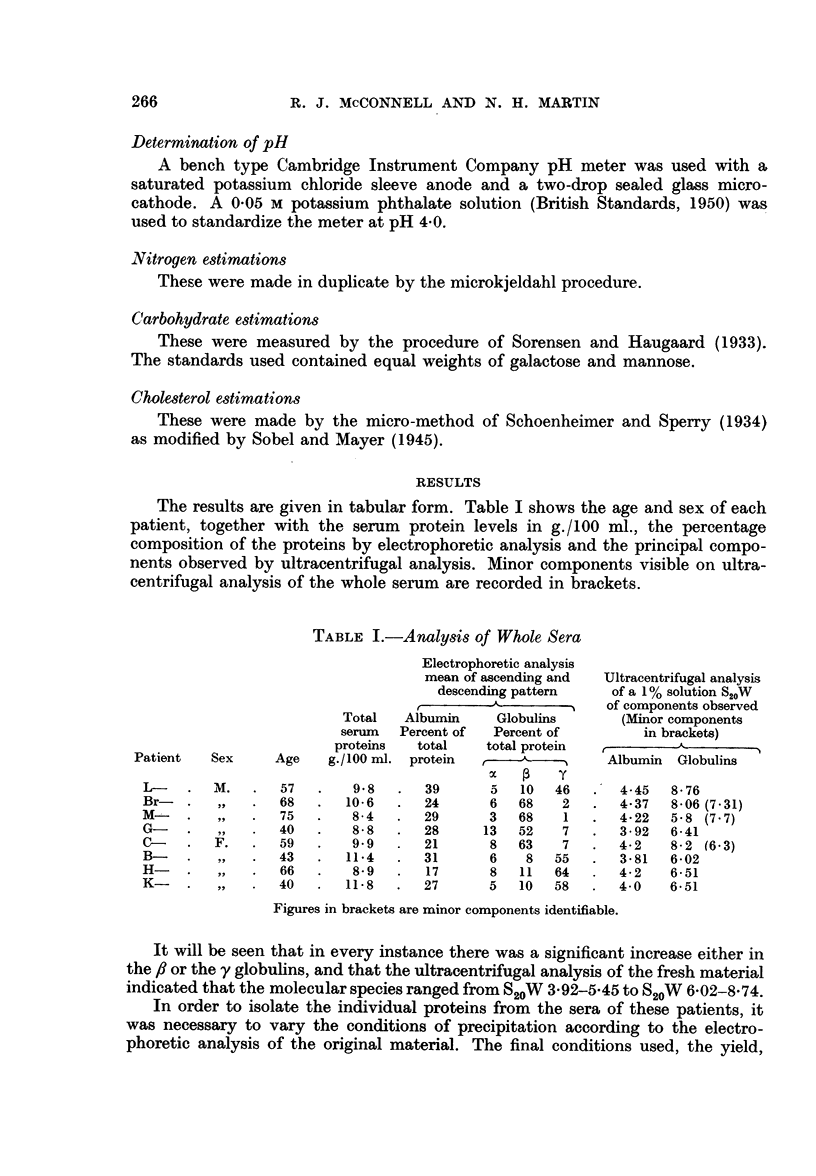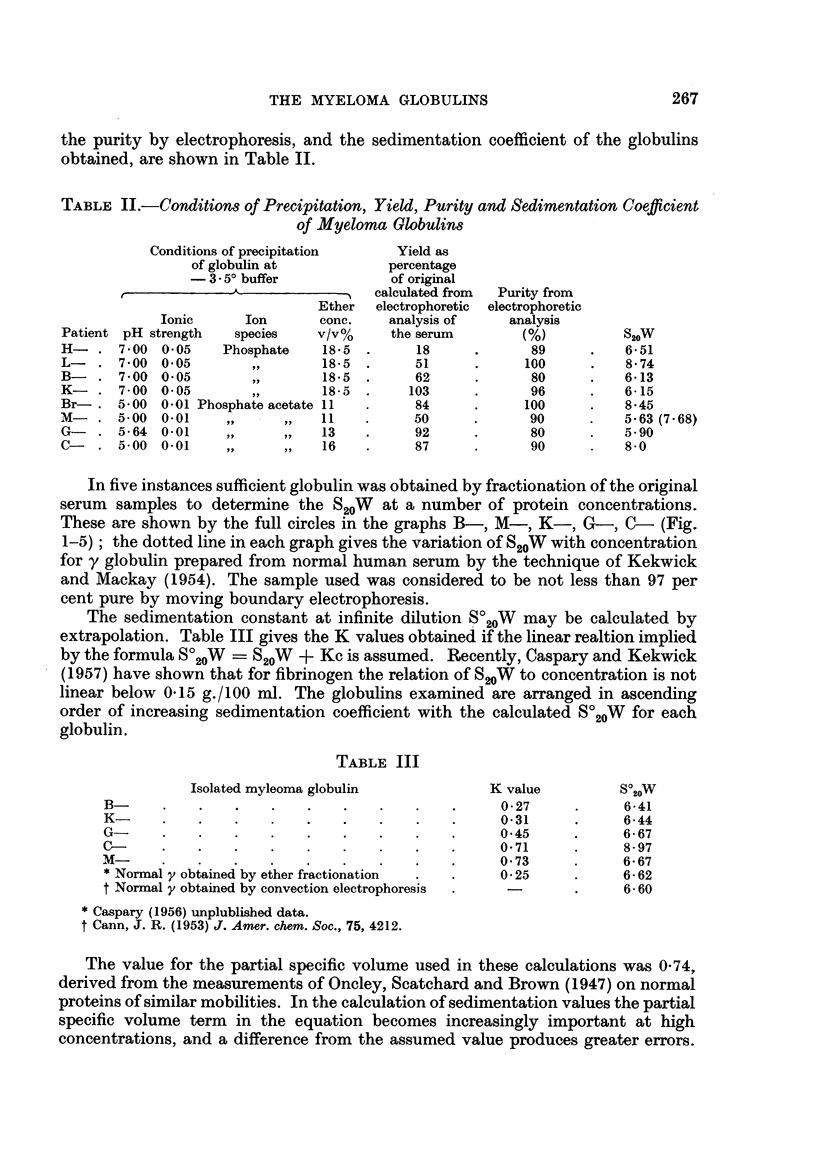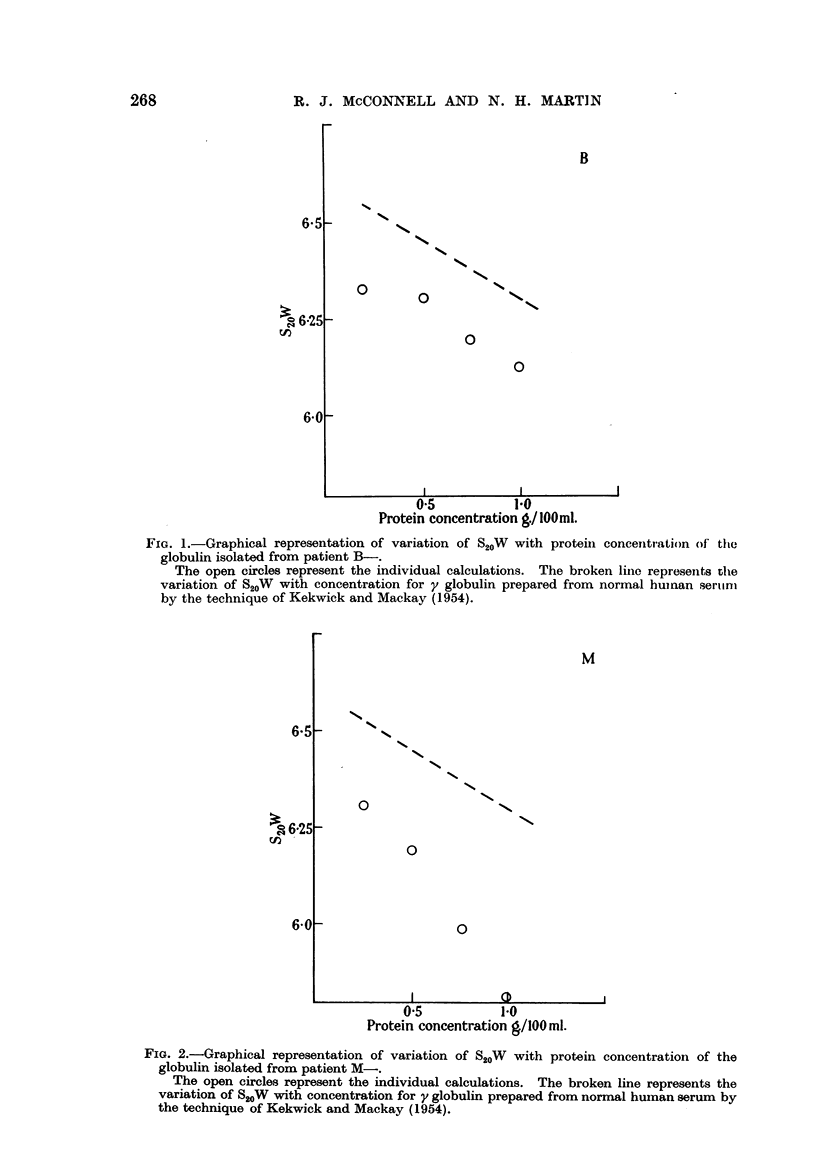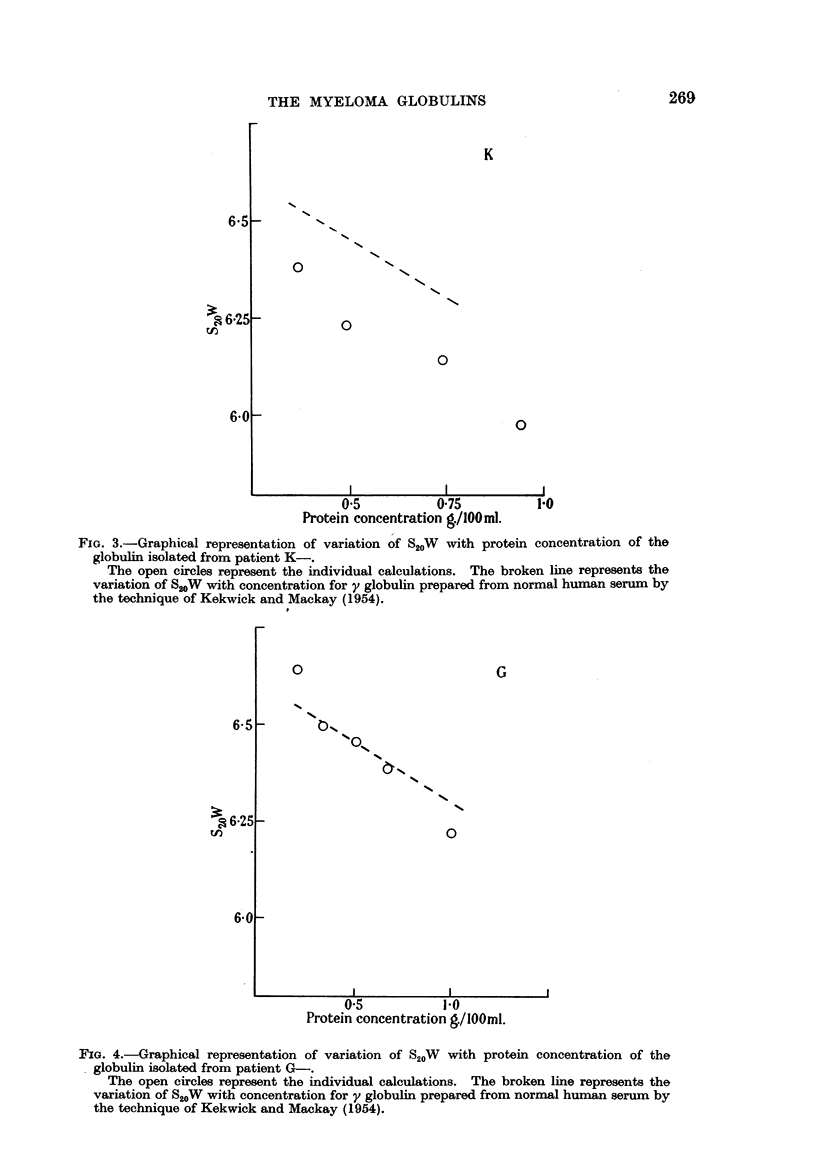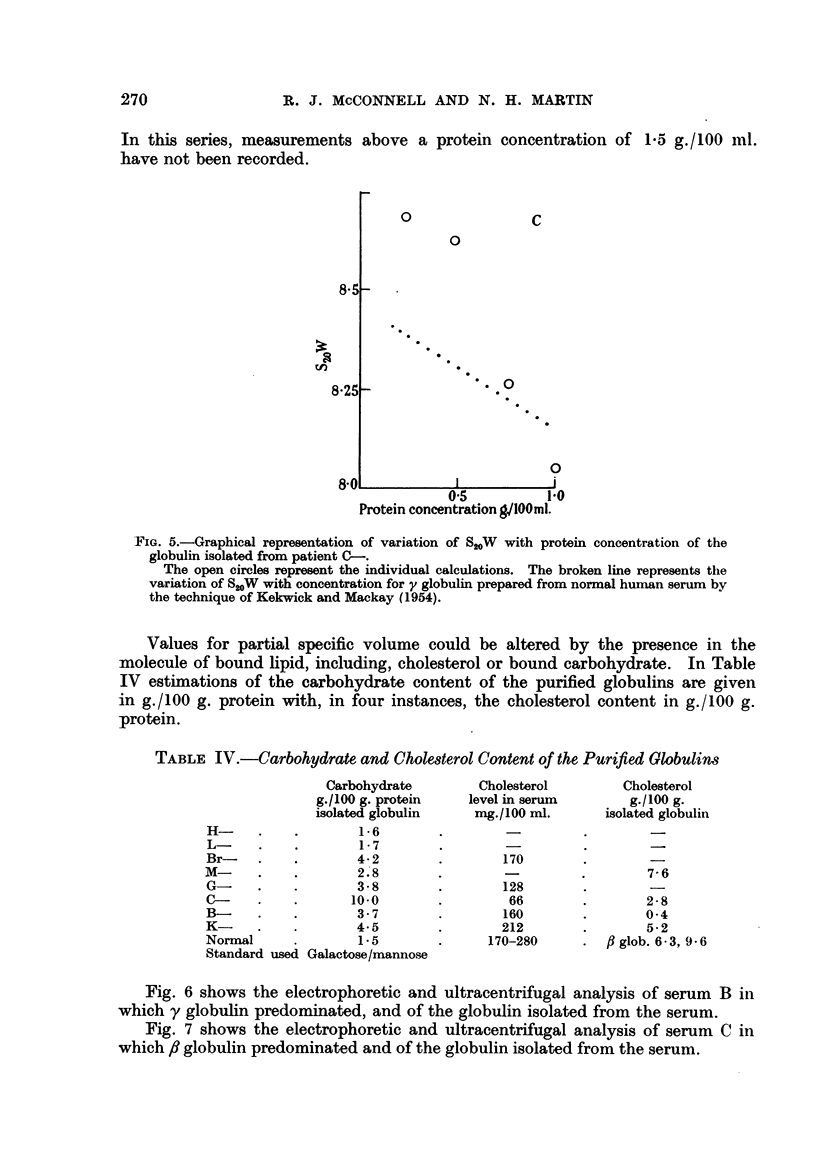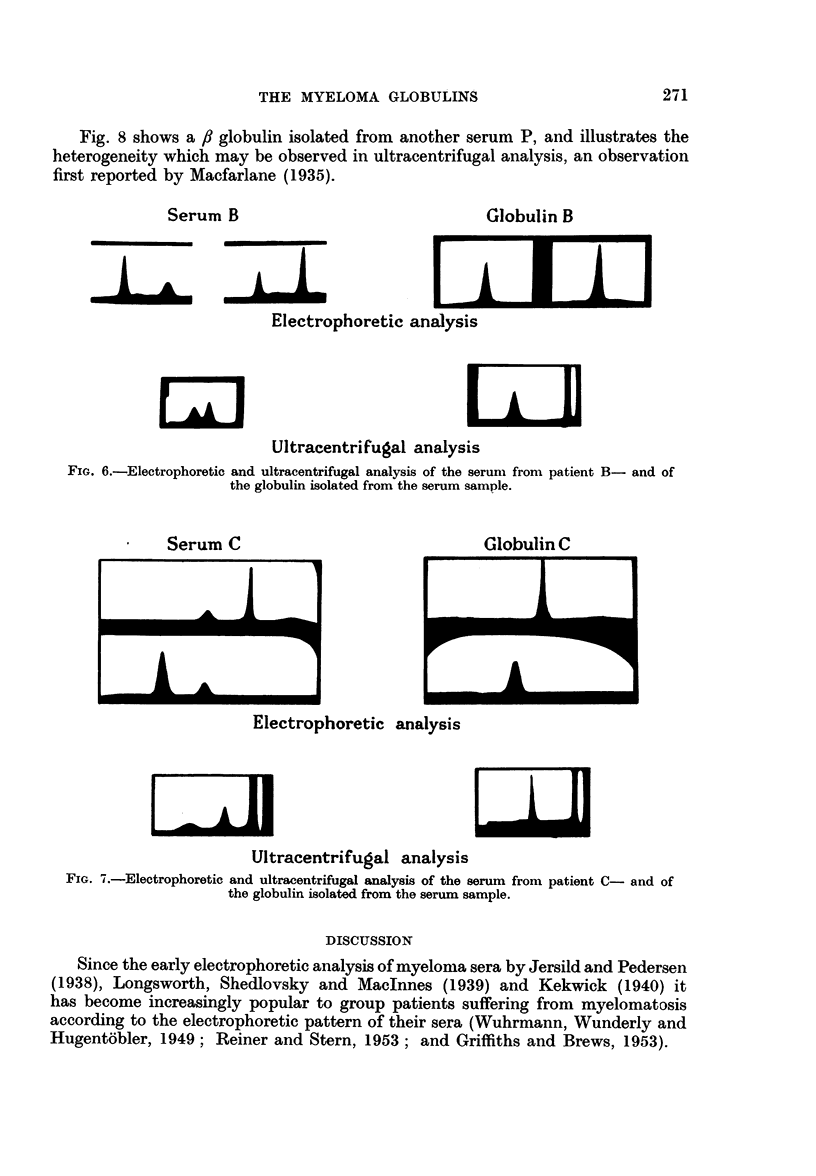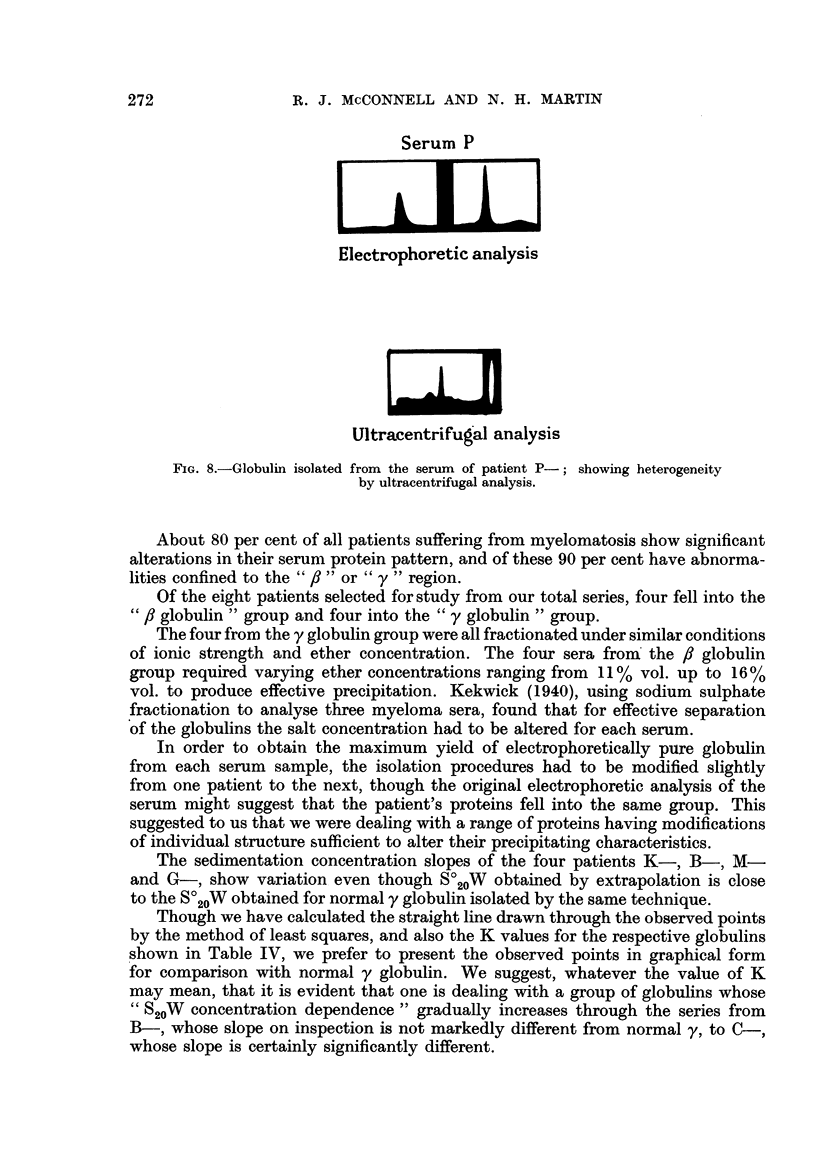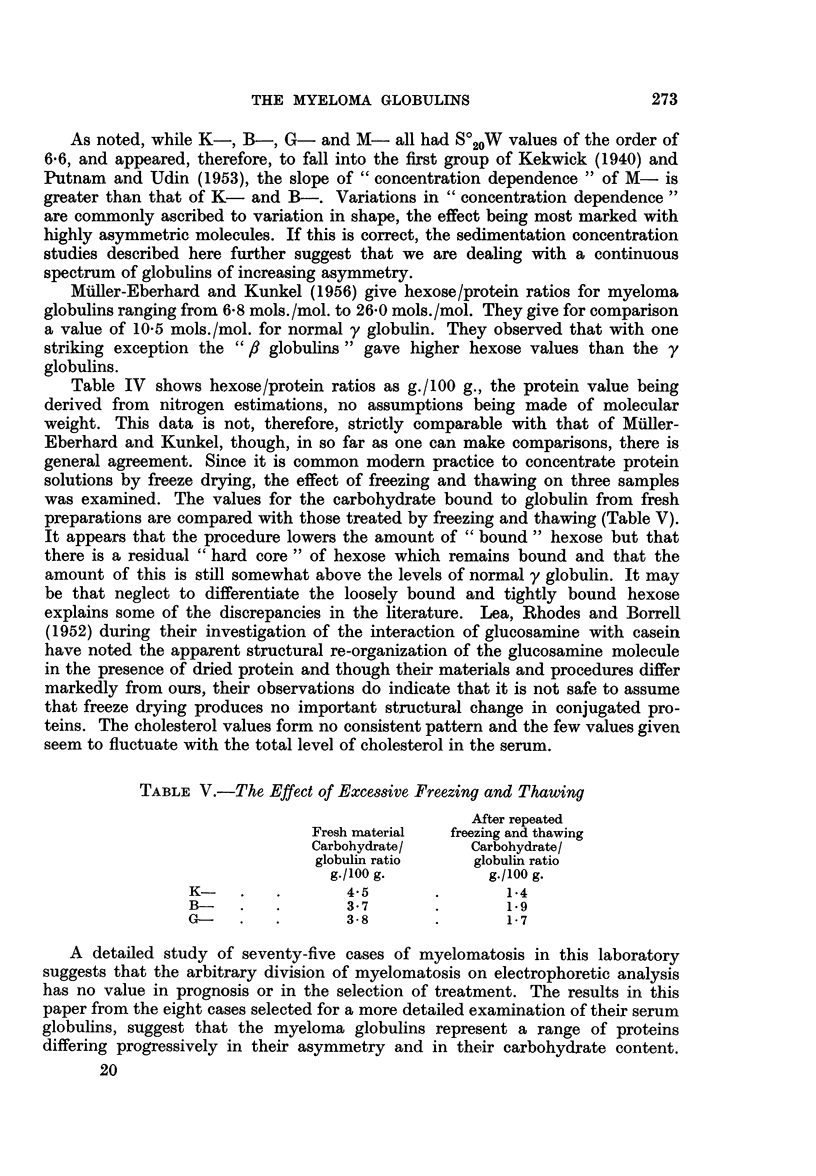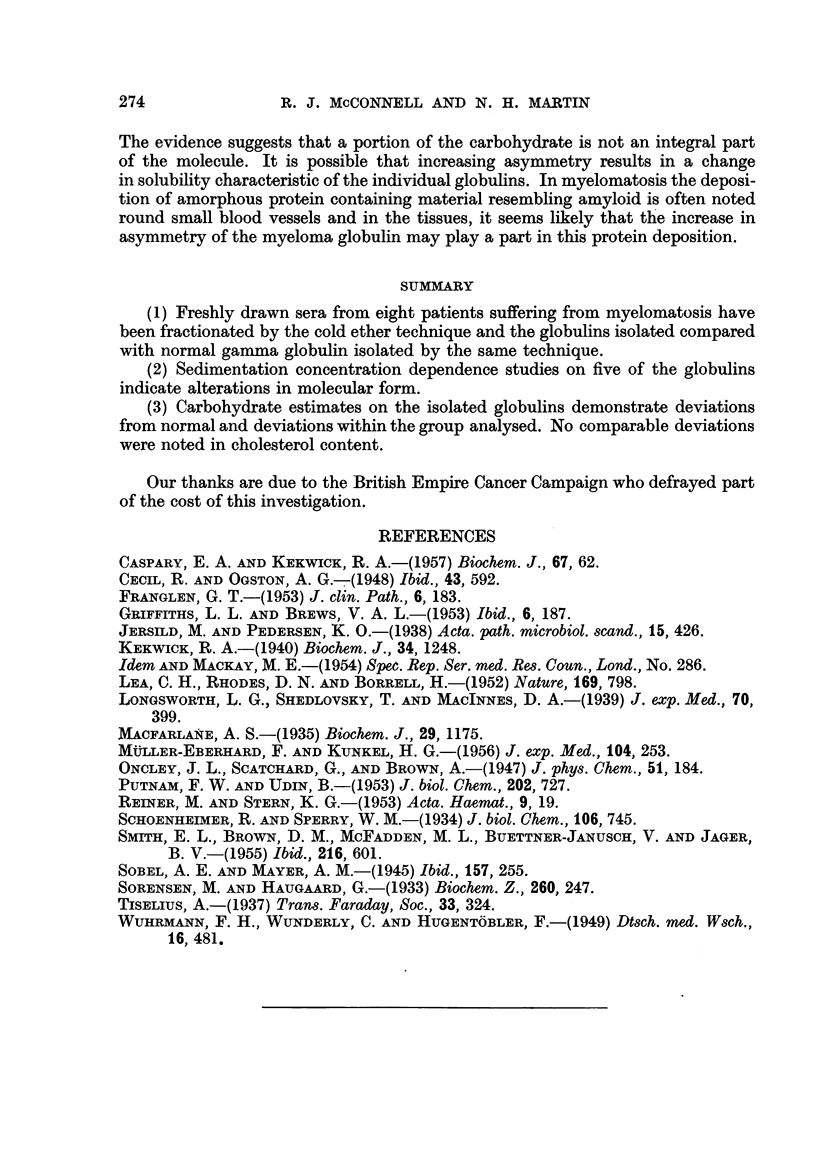# The Myeloma Globulins

**DOI:** 10.1038/bjc.1958.32

**Published:** 1958-06

**Authors:** R. J. McConnell, N. H. Martin


### THE MYELOMA GLOBULINS

R. J. McCONNELL AND N. H. MARTIN

*From the Department of Chemical Pathology, St. George's Hospital, London, S.W.1*

Received for publication March 28, 1958

EVER since Macfarlane (1935) examined in the ultracentrifuge the sera of two patients suffering from myelomatosis and demonstrated the presence of abnormal globulins in one, the plasma protein disturbances in this disease have been the subject of physico-chemical analysis.

Kekwick (1940) examined sera from five patients and found in one, as Macfarlane had found, components not found ordinarily in the sera from normal persons. In addition he examined the sera by the Tiselius technique of electrophoresis and observed that the specimen which contained the abnormal components by ultracentrifugal analysis contained an excess of $\beta$ globulins, whereas the other sera in which no abnormal components were demonstrated, contained excesses of $\gamma$ globulins.

Kekwick used salt fractionation to isolate both the myeloma $\beta$ and $\gamma$ globulins and compared them with preparations of normal $\gamma$ globulin.

Similar behaviour was observed in one serum examined by Putnam and Udin (1953) using a variety of techniques for the isolation of the globulins.

Kekwick (1940) and, later, Smith, Brown, McFadden, Buettner-Janusch and Jager (1955) using the orcinol technique, found that the $\beta$ myeloma globulins they examined contained more carbohydrate than the $\gamma$ myeloma globulins, and differed in their iso-electric point. Smith and his associates (1955) suggested that this difference might be associated with variations in hexuronic acid.

In 1954 Kekwick and Mackay described a detailed procedure for low temperature bulk fractionation of serum proteins using ether. The method was primarily designed to separate $\gamma$ globulins with minimal disturbance of their physico-chemical behaviour and biological activity.

In the following investigation of the myeloma globulins, isolation from the original serum samples was achieved by adapting the Kekwick and Mackay procedure to small quantities.

The globulins separated have been compared with $\gamma$ globulin from pooled normal serum prepared in the same way.

### CLINICAL MATERIAL AND ANALYTICAL METHODS

Eight patients were selected from a larger series, who had been under careful clinical observation for some time. Repeated electrophoretic analyses of their sera showed consistent individual abnormalities. Fresh blood samples were drawn from the antecubital veins of the selected patients without occlusion. Twenty to thirty ml. of blood was collected into sterile glass bottles and allowed to clot at 2° C. and the serum stored at — 2° C. The patients were fasted for twelve hours before the collection and were at complete rest.

*Fractionation procedures*

In essential principles, the procedures adopted were those described in detail by Kekwick and Mackay (1954).

Since the starting material was serum and not citrated plasma the initial step precipitating fibrinogen was omitted, and the crude globulins precipitated from a solution adjusted to pH 5·5–5·7, $I = 0{\cdot}038$ by the addition of 18·5 vol. % of ether at — 3·5°. These globulins were then redissolved at pH 4·0 in acetate-phosphate buffer, brought to pH 6·0 at an ionic strength 0·01 at 0° C, and reprecipitated by addition of ether concentration between 9–18 vol. % depending upon the characteristics of the particular globulins, with the temperature reduced to — 3·5°. Kekwick and Mackay (1954) show that the composition of the precipitate of $\beta$ globulins from normal serum is particularly sensitive to varying concentrations of ether. The same sensitivity to ether was observed in the range of globulins separated from myeloma sera.

The fractionation of myeloma sera rich in $\gamma$ globulins by the technique of Kekwick and Mackay, on the small scales dictated by the amount of material available, gave yields of the final product that were often very poor, sometimes falling as low as 18 per cent of the calculated total $\gamma$ globulin content of the original serum, compared with 80 per cent from samples of normal serum.

In order to increase the yield by reducing manipulation and, avoiding loss of $\gamma$ globulin to other fractions, a one-stage fractionation procedure was attempted, modelled on the technique of Kekwick and Mackay. It was felt that this step was justified because of the grossly abnormal distribution of proteins in the serum of patients suffering from myelomatosis.

The procedure consisted of diluting the fresh serum at 0° C. to a protein concentration of 0·3 g./100 ml. with sodium phosphate buffer pH 7·0 ionic strength 0·05. Ether was then added slowly, the temperature at the same time being lowered to — 3·5° C., until an ether concentration of 18·5 vol. had been reached. The precipitate which formed was then collected by cold centrifugation.

*Electrophoretic measurements*

(*a*) To conserve material, analysis of samples obtained during microfractionation was carried out by filter paper electrophoresis as described by Franglen (1953).

(*b*) The starting material and the final product were analysed in the Tiselius apparatus (1937) at 0°. Samples for analysis were dialysed to equilibrium at 2° against phosphate buffer pH 8·0 $I = 0{\cdot}2$.

*Ultracentrifugal analysis*

Dialysed samples in sodium phosphate buffer pH 8·0, $I = 0{\cdot}2$ in 0·15 M sodium chloride were subjected to 240,000 g. in the Svedberg oil turbine centrifuge. The protein concentration of the solution was computed from nitrogen estimations using a conversion factor of 6·24. Sedimentation coefficients were computed by the method of Cecil and Ogston (1948). The slope was calculated, using the method of least squares, from the regression of the distance moved against time with a correction for the viscosity of the solvent calculations from five or more measurements given to two places of decimals.

*Determination of pH*

A bench type Cambridge Instrument Company pH meter was used with a saturated potassium chloride sleeve anode and a two-drop sealed glass microcathode. A 0·05 M potassium phthalate solution (British Standards, 1950) was used to standardize the meter at pH 4·0.

*Nitrogen estimations*

These were made in duplicate by the microkjeldahl procedure.

*Carbohydrate estimations*

These were measured by the procedure of Sorensen and Haugaard (1933). The standards used contained equal weights of galactose and mannose.

*Cholesterol estimations*

These were made by the micro-method of Schoenheimer and Sperry (1934) as modified by Sobel and Mayer (1945).

RESULTS

The results are given in tabular form. Table I shows the age and sex of each patient, together with the serum protein levels in g./100 ml., the percentage composition of the proteins by electrophoretic analysis and the principal components observed by ultracentrifugal analysis. Minor components visible on ultracentrifugal analysis of the whole serum are recorded in brackets.

TABLE I.—*Analysis of Whole Sera*

| | | | | Electrophoretic analysis mean of ascending and descending pattern | | | | Ultracentrifugal analysis of a 1% solution $S_{20}W$ of components observed (Minor components in brackets) | |
|---|---|---|---|---|---|---|---|---|---|
| | | | Total serum proteins | Albumin Percent of total | Globulins Percent of total protein | | | | |
| Patient | Sex | Age | g./100 ml. | protein | α | β | γ | Albumin | Globulins |
| L— | M. | 57 | 9·8 | 39 | 5 | 10 | 46 | 4·45 | 8·76 |
| Br— | ,, | 68 | 10·6 | 24 | 6 | 68 | 2 | 4·37 | 8·06 (7·31) |
| M— | ,, | 75 | 8·4 | 29 | 3 | 68 | 1 | 4·22 | 5·8 (7·7) |
| G— | ,, | 40 | 8·8 | 28 | 13 | 52 | 7 | 3·92 | 6·41 |
| C— | F. | 59 | 9·9 | 21 | 8 | 63 | 7 | 4·2 | 8·2 (6·3) |
| B— | ,, | 43 | 11·4 | 31 | 6 | 8 | 55 | 3·81 | 6·02 |
| H— | ,, | 66 | 8·9 | 17 | 8 | 11 | 64 | 4·2 | 6·51 |
| K— | ,, | 40 | 11·8 | 27 | 5 | 10 | 58 | 4·0 | 6·51 |

Figures in brackets are minor components identifiable.

It will be seen that in every instance there was a significant increase either in the $\beta$ or the $\gamma$ globulins, and that the ultracentrifugal analysis of the fresh material indicated that the molecular species ranged from $S_{20}W$ 3·92–5·45 to $S_{20}W$ 6·02–8·74.

In order to isolate the individual proteins from the sera of these patients, it was necessary to vary the conditions of precipitation according to the electrophoretic analysis of the original material. The final conditions used, the yield, the purity by electrophoresis, and the sedimentation coefficient of the globulins obtained, are shown in Table II.

TABLE II.—*Conditions of Precipitation, Yield, Purity and Sedimentation Coefficient of Myeloma Globulins*

| Patient | Conditions of precipitation of globulin at — 3·5° buffer: pH | Ionic strength | Ion species | Ether conc. v/v% | Yield as percentage of original calculated from electrophoretic analysis of the serum | Purity from electrophoretic analysis (%) | $S_{20}W$ |
|---|---|---|---|---|---|---|---|
| H— | 7·00 | 0·05 | Phosphate | 18·5 | 18 | 89 | 6·51 |
| L— | 7·00 | 0·05 | „ | 18·5 | 51 | 100 | 8·74 |
| B— | 7·00 | 0·05 | „ | 18·5 | 62 | 80 | 6·13 |
| K— | 7·00 | 0·05 | „ | 18·5 | 103 | 96 | 6·15 |
| Br— | 5·00 | 0·01 | Phosphate acetate | 11 | 84 | 100 | 8·45 |
| M— | 5·00 | 0·01 | „ „ | 11 | 50 | 90 | 5·63 (7·68) |
| G— | 5·64 | 0·01 | „ „ | 13 | 92 | 80 | 5·90 |
| C— | 5·00 | 0·01 | „ „ | 16 | 87 | 90 | 8·0 |

In five instances sufficient globulin was obtained by fractionation of the original serum samples to determine the $S_{20}W$ at a number of protein concentrations. These are shown by the full circles in the graphs B—, M—, K—, G—, C— (Fig. 1–5) ; the dotted line in each graph gives the variation of $S_{20}W$ with concentration for $\gamma$ globulin prepared from normal human serum by the technique of Kekwick and Mackay (1954). The sample used was considered to be not less than 97 per cent pure by moving boundary electrophoresis.

The sedimentation constant at infinite dilution $S^{\circ}_{20}W$ may be calculated by extrapolation. Table III gives the K values obtained if the linear realtion implied by the formula $S^{\circ}_{20}W = S_{20}W + Kc$ is assumed. Recently, Caspary and Kekwick (1957) have shown that for fibrinogen the relation of $S_{20}W$ to concentration is not linear below 0·15 g./100 ml. The globulins examined are arranged in ascending order of increasing sedimentation coefficient with the calculated $S^{\circ}_{20}W$ for each globulin.

TABLE III

| Isolated myleoma globulin | K value | $S^{\circ}_{20}W$ |
|---|---|---|
| B— | 0·27 | 6·41 |
| K— | 0·31 | 6·44 |
| G— | 0·45 | 6·67 |
| C— | 0·71 | 8·97 |
| M— | 0·73 | 6·67 |
| * Normal $\gamma$ obtained by ether fractionation | 0·25 | 6·62 |
| † Normal $\gamma$ obtained by convection electrophoresis | — | 6·60 |

* Caspary (1956) unplublished data.
† Cann, J. R. (1953) *J. Amer. chem. Soc.*, **75**, 4212.

The value for the partial specific volume used in these calculations was 0·74, derived from the measurements of Oncley, Scatchard and Brown (1947) on normal proteins of similar mobilities. In the calculation of sedimentation values the partial specific volume term in the equation becomes increasingly important at high concentrations, and a difference from the assumed value produces greater errors.

FIG. 1.—Graphical representation of variation of $S_{20}W$ with protein concentration of the globulin isolated from patient B—.

The open circles represent the individual calculations. The broken line represents the variation of $S_{20}W$ with concentration for $\gamma$ globulin prepared from normal human serum by the technique of Kekwick and Mackay (1954).

FIG. 2.—Graphical representation of variation of $S_{20}W$ with protein concentration of the globulin isolated from patient M—.

The open circles represent the individual calculations. The broken line represents the variation of $S_{20}W$ with concentration for $\gamma$ globulin prepared from normal human serum by the technique of Kekwick and Mackay (1954).

FIG. 3.—Graphical representation of variation of $S_{20}W$ with protein concentration of the globulin isolated from patient K—.

The open circles represent the individual calculations. The broken line represents the variation of $S_{20}W$ with concentration for $\gamma$ globulin prepared from normal human serum by the technique of Kekwick and Mackay (1954).

FIG. 4.—Graphical representation of variation of $S_{20}W$ with protein concentration of the globulin isolated from patient G—.

The open circles represent the individual calculations. The broken line represents the variation of $S_{20}W$ with concentration for $\gamma$ globulin prepared from normal human serum by the technique of Kekwick and Mackay (1954).

In this series, measurements above a protein concentration of 1·5 g./100 ml. have not been recorded.

FIG. 5.—Graphical representation of variation of $S_{20}W$ with protein concentration of the globulin isolated from patient C—.

The open circles represent the individual calculations. The broken line represents the variation of $S_{20}W$ with concentration for $\gamma$ globulin prepared from normal human serum by the technique of Kekwick and Mackay (1954).

Values for partial specific volume could be altered by the presence in the molecule of bound lipid, including, cholesterol or bound carbohydrate. In Table IV estimations of the carbohydrate content of the purified globulins are given in g./100 g. protein with, in four instances, the cholesterol content in g./100 g. protein.

TABLE IV.—*Carbohydrate and Cholesterol Content of the Purified Globulins*

| | Carbohydrate g./100 g. protein isolated globulin | Cholesterol level in serum mg./100 ml. | Cholesterol g./100 g. isolated globulin |
|---|---|---|---|
| H— | 1·6 | — | — |
| L— | 1·7 | — | — |
| Br— | 4·2 | 170 | — |
| M— | 2·8 | — | 7·6 |
| G— | 3·8 | 128 | — |
| C— | 10·0 | 66 | 2·8 |
| B— | 3·7 | 160 | 0·4 |
| K— | 4·5 | 212 | 5·2 |
| Normal | 1·5 | 170–280 | $\beta$ glob. 6·3, 9·6 |
| Standard used | Galactose/mannose | | |

Fig. 6 shows the electrophoretic and ultracentrifugal analysis of serum B in which $\gamma$ globulin predominated, and of the globulin isolated from the serum.

Fig. 7 shows the electrophoretic and ultracentrifugal analysis of serum C in which $\beta$ globulin predominated and of the globulin isolated from the serum.

Fig. 8 shows a $\beta$ globulin isolated from another serum P, and illustrates the heterogeneity which may be observed in ultracentrifugal analysis, an observation first reported by Macfarlane (1935).

FIG. 6.—Electrophoretic and ultracentrifugal analysis of the serum from patient B— and of the globulin isolated from the serum sample.

FIG. 7.—Electrophoretic and ultracentrifugal analysis of the serum from patient C— and of the globulin isolated from the serum sample.

### DISCUSSION

Since the early electrophoretic analysis of myeloma sera by Jersild and Pedersen (1938), Longsworth, Shedlovsky and MacInnes (1939) and Kekwick (1940) it has become increasingly popular to group patients suffering from myelomatosis according to the electrophoretic pattern of their sera (Wuhrmann, Wunderly and Hugentöbler, 1949 ; Reiner and Stern, 1953 ; and Griffiths and Brews, 1953).

FIG. 8.—Globulin isolated from the serum of patient P— ; showing heterogeneity by ultracentrifugal analysis.

About 80 per cent of all patients suffering from myelomatosis show significant alterations in their serum protein pattern, and of these 90 per cent have abnormalities confined to the " $\beta$ " or " $\gamma$ " region.

Of the eight patients selected for study from our total series, four fell into the " $\beta$ globulin " group and four into the " $\gamma$ globulin " group.

The four from the $\gamma$ globulin group were all fractionated under similar conditions of ionic strength and ether concentration. The four sera from the $\beta$ globulin group required varying ether concentrations ranging from 11% vol. up to 16% vol. to produce effective precipitation. Kekwick (1940), using sodium sulphate fractionation to analyse three myeloma sera, found that for effective separation of the globulins the salt concentration had to be altered for each serum.

In order to obtain the maximum yield of electrophoretically pure globulin from each serum sample, the isolation procedures had to be modified slightly from one patient to the next, though the original electrophoretic analysis of the serum might suggest that the patient's proteins fell into the same group. This suggested to us that we were dealing with a range of proteins having modifications of individual structure sufficient to alter their precipitating characteristics.

The sedimentation concentration slopes of the four patients K—, B—, M— and G—, show variation even though $S°_{20}W$ obtained by extrapolation is close to the $S°_{20}W$ obtained for normal $\gamma$ globulin isolated by the same technique.

Though we have calculated the straight line drawn through the observed points by the method of least squares, and also the K values for the respective globulins shown in Table IV, we prefer to present the observed points in graphical form for comparison with normal $\gamma$ globulin. We suggest, whatever the value of K may mean, that it is evident that one is dealing with a group of globulins whose " $S_{20}W$ concentration dependence " gradually increases through the series from B—, whose slope on inspection is not markedly different from normal $\gamma$, to C—, whose slope is certainly significantly different.

As noted, while K—, B—, G— and M— all had $S^{\circ}_{20}W$ values of the order of 6·6, and appeared, therefore, to fall into the first group of Kekwick (1940) and Putnam and Udin (1953), the slope of " concentration dependence " of M— is greater than that of K— and B—. Variations in " concentration dependence " are commonly ascribed to variation in shape, the effect being most marked with highly asymmetric molecules. If this is correct, the sedimentation concentration studies described here further suggest that we are dealing with a continuous spectrum of globulins of increasing asymmetry.

Müller-Eberhard and Kunkel (1956) give hexose/protein ratios for myeloma globulins ranging from 6·8 mols./mol. to 26·0 mols./mol. They give for comparison a value of 10·5 mols./mol. for normal $\gamma$ globulin. They observed that with one striking exception the " $\beta$ globulins " gave higher hexose values than the $\gamma$ globulins.

Table IV shows hexose/protein ratios as g./100 g., the protein value being derived from nitrogen estimations, no assumptions being made of molecular weight. This data is not, therefore, strictly comparable with that of Müller-Eberhard and Kunkel, though, in so far as one can make comparisons, there is general agreement. Since it is common modern practice to concentrate protein solutions by freeze drying, the effect of freezing and thawing on three samples was examined. The values for the carbohydrate bound to globulin from fresh preparations are compared with those treated by freezing and thawing (Table V). It appears that the procedure lowers the amount of " bound " hexose but that there is a residual " hard core " of hexose which remains bound and that the amount of this is still somewhat above the levels of normal $\gamma$ globulin. It may be that neglect to differentiate the loosely bound and tightly bound hexose explains some of the discrepancies in the literature. Lea, Rhodes and Borrell (1952) during their investigation of the interaction of glucosamine with casein have noted the apparent structural re-organization of the glucosamine molecule in the presence of dried protein and though their materials and procedures differ markedly from ours, their observations do indicate that it is not safe to assume that freeze drying produces no important structural change in conjugated proteins. The cholesterol values form no consistent pattern and the few values given seem to fluctuate with the total level of cholesterol in the serum.

TABLE V.—*The Effect of Excessive Freezing and Thawing*

| | Fresh material Carbohydrate/ globulin ratio g./100 g. | After repeated freezing and thawing Carbohydrate/ globulin ratio g./100 g. |
|---|---|---|
| K— | 4·5 | 1·4 |
| B— | 3·7 | 1·9 |
| G— | 3·8 | 1·7 |

A detailed study of seventy-five cases of myelomatosis in this laboratory suggests that the arbitrary division of myelomatosis on electrophoretic analysis has no value in prognosis or in the selection of treatment. The results in this paper from the eight cases selected for a more detailed examination of their serum globulins, suggest that the myeloma globulins represent a range of proteins differing progressively in their asymmetry and in their carbohydrate content.

The evidence suggests that a portion of the carbohydrate is not an integral part of the molecule. It is possible that increasing asymmetry results in a change in solubility characteristic of the individual globulins. In myelomatosis the deposition of amorphous protein containing material resembling amyloid is often noted round small blood vessels and in the tissues, it seems likely that the increase in asymmetry of the myeloma globulin may play a part in this protein deposition.

### SUMMARY

(1) Freshly drawn sera from eight patients suffering from myelomatosis have been fractionated by the cold ether technique and the globulins isolated compared with normal gamma globulin isolated by the same technique.

(2) Sedimentation concentration dependence studies on five of the globulins indicate alterations in molecular form.

(3) Carbohydrate estimates on the isolated globulins demonstrate deviations from normal and deviations within the group analysed. No comparable deviations were noted in cholesterol content.

Our thanks are due to the British Empire Cancer Campaign who defrayed part of the cost of this investigation.

### REFERENCES

Caspary, E. A. and Kekwick, R. A.—(1957) *Biochem. J.*, **67**, 62.
Cecil, R. and Ogston, A. G.—(1948) *Ibid.*, **43**, 592.
Franglen, G. T.—(1953) *J. clin. Path.*, **6**, 183.
Griffiths, L. L. and Brews, V. A. L.—(1953) *Ibid.*, **6**, 187.
Jersild, M. and Pedersen, K. O.—(1938) *Acta. path. microbiol. scand.*, **15**, 426.
Kekwick, R. A.—(1940) *Biochem. J.*, **34**, 1248.
*Idem* and Mackay, M. E.—(1954) *Spec. Rep. Ser. med. Res. Coun., Lond.*, No. 286.
Lea, C. H., Rhodes, D. N. and Borrell, H.—(1952) *Nature*, **169**, 798.
Longsworth, L. G., Shedlovsky, T. and MacInnes, D. A.—(1939) *J. exp. Med.*, **70**, 399.
Macfarlane, A. S.—(1935) *Biochem. J.*, **29**, 1175.
Müller-Eberhard, F. and Kunkel, H. G.—(1956) *J. exp. Med.*, **104**, 253.
Oncley, J. L., Scatchard, G., and Brown, A.—(1947) *J. phys. Chem.*, **51**, 184.
Putnam, F. W. and Udin, B.—(1953) *J. biol. Chem.*, **202**, 727.
Reiner, M. and Stern, K. G.—(1953) *Acta. Haemat.*, **9**, 19.
Schoenheimer, R. and Sperry, W. M.—(1934) *J. biol. Chem.*, **106**, 745.
Smith, E. L., Brown, D. M., McFadden, M. L., Buettner-Janusch, V. and Jager, B. V.—(1955) *Ibid.*, **216**, 601.
Sobel, A. E. and Mayer, A. M.—(1945) *Ibid.*, **157**, 255.
Sorensen, M. and Haugaard, G.—(1933) *Biochem. Z.*, **260**, 247.
Tiselius, A.—(1937) *Trans. Faraday, Soc.*, **33**, 324.
Wuhrmann, F. H., Wunderly, C. and Hugentöbler, F.—(1949) *Dtsch. med. Wsch.*, **16**, 481.